# Case report: Rare variants in the *MTRR* gene, 66GG and 524TT cause hyperhomocysteinemia and folic acid deficiency linked to schizophrenia

**DOI:** 10.3389/fpsyt.2024.1353308

**Published:** 2024-07-12

**Authors:** Chih-Chia Huang

**Affiliations:** ^1^ Tsaotun Psychiatric Center, Ministry of Health and Welfare, Nantou, Taiwan; ^2^ Program in Translational Medicine, National Chung Hsing University, Taichung, Taiwan

**Keywords:** hyperhomocysteinemia, folic acid, MTRR, MSR, delayed diagnosis

## Abstract

We present an adult patient with schizophrenia who was later found to have hyperhomocysteinemia, a condition that increases the risk of several diseases, due to a deficiency in folic acid. Although folic acid supplementation quickly normalized the hyperhomocysteinemia and folic acid levels, it did not significantly improve the overall mental and cognitive health. Genotype analysis was performed and the patient was found to have two pathogenic variants in the *MTRR* gene, 66GG and 524TT, which encodes for methionine synthase reductase (MSR), an enzyme crucial for homocysteine metabolism. The results can shed light on the reasons behind the patient’s hyperhomocysteinemia and folic acid deficiency. Hyperhomocysteinemia confers an increased risk of several diseases. Indeed, the patient has neurodevelopment and cardiovascular health problems for decades. Given the rarity of the condition and the nonspecific nature of the symptoms, the detection of hyperhomocysteinemia or MSR deficiency can often be delayed or overlooked. Considering the potential irreversible and detrimental consequences of prolonged hyperhomocysteinemia and folic acid deficiency that our patient is likely experiencing, we suggest that clinicians be vigilant for associated signs when they encounter adolescents exhibiting psychotic symptoms, especially those with additional physical symptoms and a history of resistance to treatment.

## Introduction

Schizophrenia is a complex disorder with many contributing factors, including genetics (1, 2). Researchers have found that genetic variants in genes involved in vitamin B metabolism may confer susceptibility to or protective effects against development of schizophrenia (3–5).

Some genetic variants related to vitamin B metabolism can cause high levels of homocysteine, a harmful amino acid that has been linked to schizophrenia and other mental disorders (3). Previous studies have also found lower levels of folic acid, a form of vitamin B, in patients with long-term schizophrenia (6) and first-episode psychosis (7) compared with healthy controls. However, many patients with schizophrenia do not have their homocysteine and folic acid levels checked regularly, and may suffer from undiagnosed deficiencies that worsen their symptoms. But, the potential benefits of vitamin B supplementation are not well established and the exact mechanisms by which vitamin B metabolism affects schizophrenia are still unclear.

We report a patient with refractory schizophrenia and comorbidities of treatment-resistant hypertension, old infraction, and seizure who had hyperhomocysteinemia and folic acid deficiency due to two rare mutations in the *MTRR* gene, which encodes methionine synthase reductase (MSR), another key regulating enzyme involved in the folate cycle that contributes to the metabolism of homocysteine. MSR is primarily involved in the regeneration of methionine from homocysteine. Through its restoring activity, the MSR enzyme also plays a crucial role in the metabolism of homocysteine and folic acid (8–10). But, the levels of homocysteine and folic acid never checked before in the patient. Written informed consent was obtained from the patient for publication of this case report.

## Case description

Our patient is a 39-year-old man who had a learning disability since childhood. He also experienced difficulties in social relationships, which intensified when he was 16 years old. However, his motor and physical development were not delayed. Due to a progressive worsening of hallucinations, delusions, and behavioral disturbances, including aggression and destructive behaviors, he was unable to complete his senior high school education. Consequently, he was sent to a psychiatric hospital and diagnosed with schizophrenia at the age of 16. The patient’s parents divorced during his adolescence. Regarding the psychiatric family history, his mother alleges that his father was an alcoholic and has since passed away. His mother has hypertension and does not have stroke or psychiatric disease. Our patient had a history of treatment resistance to multiple antipsychotics including quetiapine, risperidone, amisulpride, haloperidol, flupentixol, valproate, and carbamazepine. His condition continued to worsen over time. In addition, he developed metabolic syndrome (height 172.8 cm, weight 112.8 kg). Hypertension was found at 3 years ago. But, even with combination therapy of three antihypertensive drugs (hydralazine at 300 mg daily, captopril at 25 mg daily, and amlodipine/valsartan/hydrochlorothiazide at 5/160/12.5 mg daily), the hypertension remains significant (SBP > 160, DBP > 110). He does not consume alcohol, however, he does smoke between 4 to 6 cigarettes daily. In addition, the Wechsler Adult Intelligence Scale, third edition, reported a borderline intellectual disability with a Full-Scale Intelligence Quotient (FIQ) of 66, a Verbal Intelligence Quotient (VIQ) of 75, and a Performance Intelligence Quotient (PIQ) of 56. Haloperidol (20 mg daily) and carbamazepine (600 mg daily) were prescribed at least 1 year prior to this admission. Despite the patient’s favorable drug compliance, his agitation and psychotic symptoms were exacerbated with referential delusions, persecutory delusions, and behavioral disturbances. He frequently threatened his family members under delusion of theft. He would suddenly become aggressive, both verbally and physically; subsequently, he was admitted.

**Figure 1:**
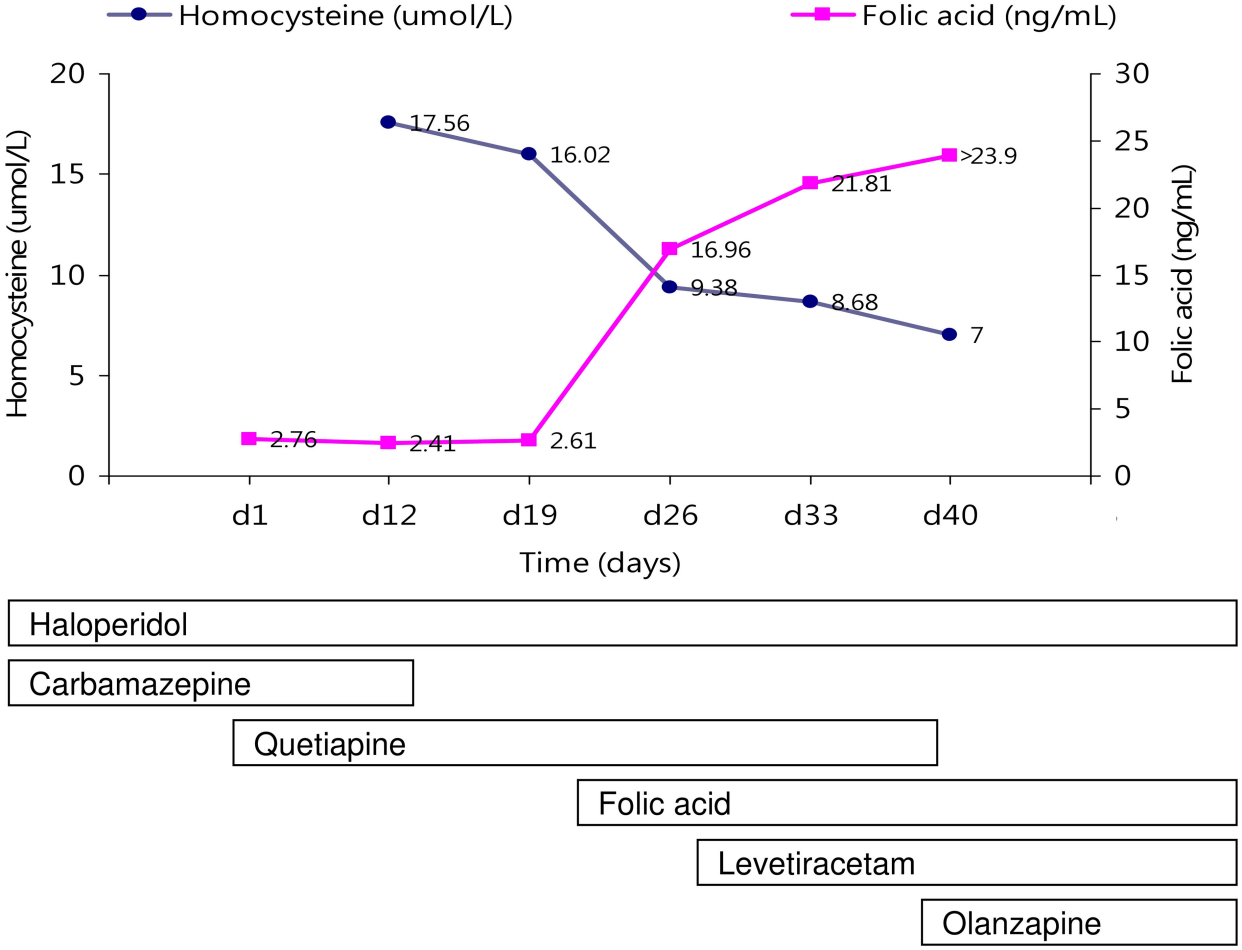
The changes of serum hemocysteine and folic acid levels with neuropsychiatric treatments are indicated.

After admission, physical examination and routine laboratory tests (biochemistry, lipid profile, fasting glucose, vitamin B12 level, folic acid level, blood carbamazepine level, total blood count) were performed. Hypertriglyceridemia (163 mg/dL), hyperglycemia (fasting blood sugar: 117 mg/dL, HbA1c: 6.4%), and folic acid deficiency (2.76 ng/ml) were noted. There is no anemia. Moreover, treatment with haloperidol and carbamazepine was shifted to that with quetiapine and carbamazepine. Because carbamazepine is known to cause folic acid deficiency through the induction of cytochrome P450 leading to hyperhomocysteinemia (11), carbamazepine at 600 mg daily was tapered down to 400 mg daily, then was discontinued (**Figure 1**). But, even the carbamazepine has been discontinued for 1 week, folic acid deficiency and hyperhomocysteinemia were persistent (homocysteine:17.56 μmol/liter, 16.02 μmol/liter; folic acid:2.41 ng/ml, 2.61 ng/ml). And, folic acid supplement (5 mg daily) was provided. With these treatments, the patient’s folic acid deficiency and hyperhomocysteinemia soon subsided (homocysteine:7μmol/liter, folic acid: >23.90 ng/ml). In addition, after combination therapy of haloperidol (10 mg daily) and quetiapine (450 mg daily), the patient reported he had felt improvement in his sleep quality. But, following the 14 days discontinuous of carbamazepine, the patient had a tonic-clonic seizure at the 26^th^ day of hospitalization. According to the statement of family members, he had no prior history of seizures. Both brain computed tomography scan and electroencephalography were arranged, and an old infarction over the right cerebellar region, prominent beta activities superimposed on the background in the whole brain area, and transient theta activity were identified. After the patient’s seizure, the quetiapine dose was decreased to 200 mg daily and levetiracetam (1000 mg daily) was added; haloperidol (10 mg daily) administration was maintained. But, progressively experienced severe psychiatric decompensation with related uncontrollable behavioral disturbances was noted. The patient claimed he felt very furious because his belongings were stolen. He also was very upset about the worsening of his insomnia. Because of an exacerbation of psychotic symptoms, quetiapine was replaced by olanzapine, and the dosage of olanzapine was titrated to 20 mg daily. The severity of psychotic symptoms was alleviated a little and no seizure occurred. However, gradual body weight gain was observed.

## Discussions

Our patient had a history of early-onset psychosis and hypertension as well as refractoriness to antipsychotic and antihypertension agents for decades. These are important clues enabling an early diagnosis of hyperhomocysteinemia and folic acid deficiency. Some reports have suggested that patients with schizophrenia who have abnormal homocysteine or vitamin levels in the blood may respond to adjunctive vitamin supplementation (5); which would be helpful for treatment initiation to reduce the morbidity and mortality related to this disorder. Our patient was found to have hyperhomocysteinemia and folic acid deficiency after a delay of several decades. For our patient, the supplementation of folic acid and vitamin B rapidly normalized his homocysteine and folic acid levels, but no significant change was noted in his psychotic symptoms.

Several psychiatric and neuro-developmental diseases have been linked to vitamin B deficiency in clinical, epidemiological, and genetic studies (12–15). Among related deficiencies, methylenetetrahydrofolate reductase (MTHFR) has attracted the most interest. Individuals with MTHFR deficiency often exhibit psychiatric manifestations (16, 17). A case of MTHFR deficiency precipitated by antiepileptic drug administration has been reported (18). Individuals with the commonest *MTHFR* variant, the 677T allele, have reductions in enzyme activity of 27% to 78% (19, 20). The second most common *MTHFR* variant, the 1298 C allele, reduces MTHFR activity by 8% to 40% (20–23). Thus, we examined the C667T and A1298C variants of *MTHFR*. However, the C677T and A1298C polymorphisms of *MTHFR* yielded CC and AA, respectively, which are normal variants. Because the gene analysis could not explain the hyperhomocysteinemia and folic acid deficiency, we turned our focus to MSR, another key regulating enzyme involved in the folate cycle that contributes to the metabolism of homocysteine. MSR is primarily involved in the regeneration of methionine from homocysteine. Through its restoring activity, the MSR enzyme also plays a crucial role in the metabolism of homocysteine and folic acid (8–10). In this patient, genetic analysis revealed the uncommon co-existed two pathogenic variants of the *MTRR* gene: 66GG and 524TT. MSR is encoded by the *MTRR* gene; the *MTRR* 66A>G polymorphism is the best-studied variant of this gene. In this polymorphism, methionine substitutes isoleucine at codon 22 (24). Compared with the wild type, the *MTRR* 66GG variant resulted in three times lower enzymatic activity with a four times higher homocysteine/methionine ratio in cells (25–27). In addition to the well-studied *MTRR* A66G polymorphism, another newly identified *MTRR* variant has recently been discovered, with serine substituted by leucine at position 175. It is named *MTRR* C524T, and it also affects enzymatic activity (28). A 3-fold higher ratio of MSR to methionine synthase is required for maximal activation with *MTRR* 524TT variants compared with that of the wild type enzyme (25).

Both *MTRR* 66G and 524T alleles can result in MSR enzyme deficiency; then, impaired conversion of homocysteine to methionine results in hyperhomocysteinemia. And, the long-term exposure of the patient to elevated homocysteine may cause microangiopathy over different regions of body. Herein, we report the case of a 39-year-old schizophrenia patient with the unusual characteristic of two single nucleotide polymorphisms (SNPs; 66GG and 524TT) of *MTRR* with hyperhomocysteinemia and low folic acid levels, which delayed diagnosis and treatment for 23 years. The patient described herein presented with a first manifestation of psychosis since adolescence. Later, hypertension, stroke, diabetes mellitus, and new seizure onset were noted. He had received antipsychotic agents and antihypertension drugs and exhibited poor responses at follow-up for 23 years. At our ward, he had a seizure. In addition, he was identified as having hyperhomocysteinemia and folic acid deficiency, which may result form the 66GG and 524TT polymorphisms of *MTRR*.

The variants in the *MTRR* gene 66GG is associated with an increased susceptibility to several diseases and conditions. One study has found that in a Chinese Han population, there were gender-specific interactions of *MTRR* A66G polymorphisms with overweight/obesity on serum lipid levels (29). Overweight/obese individuals who carried the *MTRR* 66GG genotype had higher serum high-density lipoprotein cholesterol levels than those with *MTRR* 66AA or AG genotypes. However, another study did not find a significant connection between the *MTRR* A66G polymorphism and being overweight/obese (30). It was discovered in one study that the *MTRR* A66G polymorphism, when combined with the *MTHFR* 677TT genotype, was linked to an increased risk of metabolic syndrome. Yet, no link was found between metabolic syndrome and *MTRR* A66G alone (31). Until now, only a few studies have analyzed *MTRR* A66G polymorphism and its association with diseases. And, the results are inconsistent. Another common polymorphism in the *MTRR* gene is the C524T. An *in vitro* study has shown that a 3-fold higher ratio of MSR to methionine synthase is needed for maximum activation with *MTRR* 524TT variant compared to the wild type enzyme (25). However, the information on the relationship between the *MTRR* C524T variant and hyperhomocysteinemia is quite inconclusive. But, studies found that the *MTRR* haplotype (66G/524C) is linked to serum osteocalcin concentrations in postmenopausal women and the development of acyanotic congenital heart diseases among Egyptian and Chinese children (28, 32). In relation to neurological conditions, some studies have been reported that *MTRR* gene 66GG was associated with spina bifida, Down syndrome, and intellectual disability (33–35). However, to our knowledge, an association between the polymorphisms of *MTRR* gene and psychotic symptoms has not been reported. But, psychosis secondary to hyperhomocysteinemia and folic acid deficiency is not uncommon (5). Vitamin B is essential for neuronal function, and polymorphisms of gene variants involved in B vitamin metabolism can result in hyperhomocysteinemia, which is linked to several psychiatric and cognitive diseases (3). Accordingly, the *MTRR* 66GG is likely involved the pathogenesis of the presented case with the unusual comorbidities of schizophrenia, hypertension, metabolic syndromes, and seizure. But, the effect of *MTRR* 524TT remains unclear. Schizophrenia is a complex disorder, and identifying a consistent association of vitamin B–related polymorphisms with psychiatric and neurological diseases remains challenging. Psychiatric and neurological diseases are heterogeneous, and most SNP alleles probably contribute negligibly to such diseases (36). The interaction of several nonsynonymous genetic variants of vitamin B–related genes may confer susceptibility to or protective effects against hyperhomocysteinemia; then, the risk of developing disease may be heightened or lowered in an individual. A large number of cases with schizophrenia should be analyzed for the synergistic effect of two pathogenic variants in the *MTRR* gene: 66GG and 524TT.

During the hospitalization, the patient had a seizure. Despite the chronic hyperhomocysteinemia and folate deficiency can contribute to neurological problems and have been linked to an increased risk of seizures (37, 38). But, the patient does not have seizure before. And, the seizure appears to have followed the discontinuation of carbamazepine. The patient’s history of carbamazepine use and its discontinuation are significant contributors to the recent seizure. However, the onset of seizures is complex and may influence by multiple factors. Our patient exhibited a combination of several factors, including discontinuation of carbamazepine, quetiapine administration, and neurodegenerative effects of the chronic hyperhomocysteinemia and folic acid deficiency caused by pathogenic genetic variants that may have increased the risk of development of seizure.

## Conclusions

This patient had psychosis and hypertension for 23 years and did not get better with medication. He came to our hospital and had a seizure. We found out that he had two genetic mutations (*MTRR* 66GG and 524TT) that caused high levels of homocysteine and low levels of folic acid in his blood. His homocysteine and folic acid levels became normal with vitamins, but his psychosis did not improve. Considering the potential irreversible and detrimental consequences of prolonged hyperhomocysteinemia and folic acid deficiency that our patient is likely experiencing, we suggest that clinicians be vigilant for associated signs. We think that giving vitamins earlier might have prevented some of the damage caused by these conditions.

The limitations of this case study should be noted. The entirety of the data was derived from the observations of one individual, and there was an absence of a control group. It’s crucial to remember that personal situations and occurrences can significantly impact the clinical results in individual cases. Specifically, folic acid levels are influenced not just by genetic variants, but also by environmental factors such as antiepileptic drugs, cigarette smoking, alcohol consumption, and obesity. The patient in question does not consume alcohol, however, he does smoke between 4 to 6 cigarettes daily. Upon admission, smoking is ceased and the antiepileptic drug carbamazepine was discontinued for 1 week before taking folic acid. However, the exact time frame to discontinue carbamazepine and cigarette smoking to avoid interaction with folic acid levels remains unknown. In addition, the patient is obese, a condition that could potentially amplify the impact of genetic variations. We can not rule out these potential effects.

## Data availability statement

The original contributions presented in the study are included in the article/supplementary materials. Further inquiries can be directed to the corresponding author.

## Ethics statement

Written informed consent was obtained from the individual(s) for the publication of any potentially identifiable images or data included in this article.

## Author contributions

CH: Writing – original draft, Writing – review & editing.
